# 2-{3-Cyano-5,5-dimethyl-4-[4-(pyrrol­idin-1-yl)buta-1,3-dien­yl]-2,5-dihydro­furan-2-yl­idene}malononitrile dichloro­methane solvate

**DOI:** 10.1107/S1600536808024719

**Published:** 2008-08-06

**Authors:** Graeme J. Gainsford, M. Delower H. Bhuiyan, Andrew J. Kay, Ward T. Robinson

**Affiliations:** a Industrial Research Limited, PO Box 31-310, Lower Hutt, New Zealand; b Department of Chemistry, University of Canterbury, Private Bag 4800, Christchurch 8140, New Zealand

## Abstract

The structure of the title compound, C_18_H_18_N_4_O·CH_2_Cl_2_, was solved using data collected from a multiple crystal (note high *R* factors). The crystal structure is dominated by two bifurcated attractive C—H⋯N(cyano) inter­actions.

## Related literature

For the synthesis, see Kay *et al.* (2004 ▶). For background, see Gainsford *et al.* (2007 ▶, 2008*a* ▶,*b* ▶,*c* ▶).
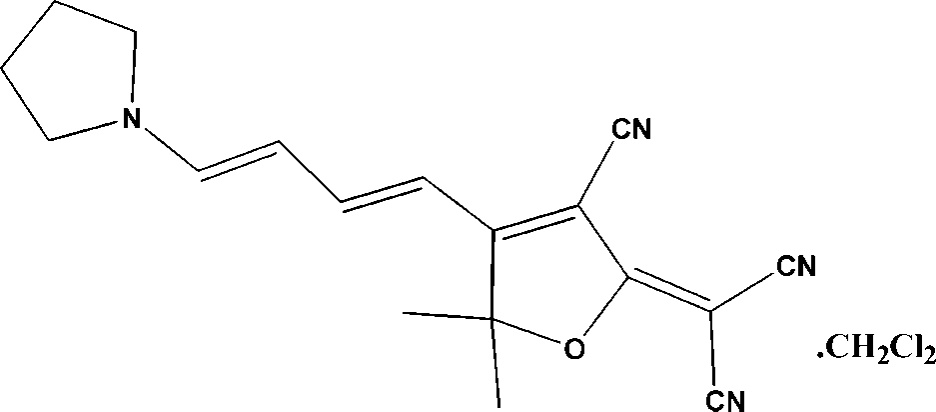


## Experimental

### 

#### Crystal data

C_18_H_18_N_4_O·CH_2_Cl_2_
*M* *_r_* = 391.29
Monoclinic, 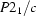

*a* = 6.8755 (8) Å
*b* = 16.8913 (17) Å
*c* = 16.6677 (18) Å
β = 93.482 (8)°
*V* = 1932.1 (4) Å^3^
*Z* = 4
Mo *K*α radiation
μ = 0.35 mm^−1^
*T* = 120 (2) K
0.30 × 0.15 × 0.13 mm

#### Data collection

Bruker–Nonius APEXII CCD area-detector diffractometer
Absorption correction: multi-scan (Blessing, 1995 ▶) *T*_min_ = 0.570, *T*_max_ = 0.955
4280 measured reflections
4280 independent reflections
2517 reflections with *I* > 2σ(*I*)
*R*_int_ = 0.099

#### Refinement

*R*[*F* ^2^ > 2σ(*F* ^2^)] = 0.095
*wR*(*F* ^2^) = 0.270
*S* = 0.99
4280 reflections
237 parameters
H-atom parameters constrained
Δρ_max_ = 1.36 e Å^−3^
Δρ_min_ = −0.47 e Å^−3^

### 

Data collection: *APEX2* (Bruker, 2005 ▶); cell refinement: *SAINT* (Bruker, 2005 ▶); data reduction: *RLATT* (Bruker, 2004 ▶), *SAINT* (Bruker, 2005 ▶) and *SADABS* (Sheldrick, 2003 ▶); program(s) used to solve structure: *SHELXS97* (Sheldrick, 2008 ▶); program(s) used to refine structure: *SHELXL97* (Sheldrick, 2008 ▶); molecular graphics: *ORTEP-3* (Farrugia, 1997 ▶) and *PLATON* (Spek, 2003 ▶); software used to prepare material for publication: *SHELXL97* and *PLATON*.

## Supplementary Material

Crystal structure: contains datablocks global, I. DOI: 10.1107/S1600536808024719/lh2664sup1.cif

Structure factors: contains datablocks I. DOI: 10.1107/S1600536808024719/lh2664Isup2.hkl

Additional supplementary materials:  crystallographic information; 3D view; checkCIF report

## Figures and Tables

**Table 1:** Hydrogen-bond geometry (Å, °)

*D*—H⋯*A*	*D*—H	H⋯*A*	*D*⋯*A*	*D*—H⋯*A*
C14—H14⋯N1^i^	0.95	2.52	3.378 (5)	150
C18—H18*B*⋯N1^i^	0.99	2.56	3.400 (5)	142

Symmetry code: (i) 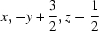.

**Fig. 1.**
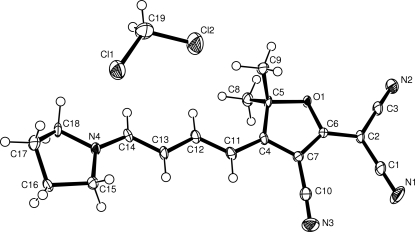
Molecular structure of the asymmetic unit (Farrugia, 1997); displacement ellipsoids are shown at the 50% probability level.

## supplementary crystallographic information

### Comment

We have previously reported on the synthesis of a number of high figure of merit chromophores for nonlinear optics (Kay *et al.*, 2004), and the X-ray crystallographic and structural properties of crucial dye precursors used (Gainsford *et al.*, 2007, 2008a,b). A closely related compound 2-[3-Cyano-5,5-dimethyl-4-(6-pyrrolidin-1-yl-hexa -1,3,5-trienyl)-5*H*-furan-2-ylidene]-malononitrile will be reported shortly (Gainsford *et al.*, 2008c).

In the crystal structure, the molecules (Fig. 1) are bound into planar dimer units *via* a polyene C–H and pyrollidine C–H bifurcated interaction with one cyano nitrogen atom (N1, Table 1). Other very weak intermolecular interactions providing inter-plane or solvent links, such as the one between the dichloromethane H19A and N1 (2.70 Å), are consistent with the difficulty found in obtaining a good single-crystal of this compound.

### Experimental

To a solution of 5.8 mmole of {4-(4-Acetanilido-*trans*-1,3-butadienyl) -3-cyano-5,5-dimethyl-2(5*H*)-furanylidene}propanedinitrile (Compound 11*b*, Kay *et al.*, 2004) in 30 ml of ethanol was added an equimolar quantity of pyrrolidine. The solution was refluxed 1 h, cooled and the product collected by filtration and washed with ethanol. λ_max_ 530 nm (pyridine); 530 nm (DMF) log_10_ε 5.22. Final crystallization was from a 1:1 dichloromethane/ ethyl acetate mixture.

### Refinement

Diffraction data was extracted from the major of multiple intersecting lattices using RLATT (Bruker, 2004). The structure was solved by direct methods but refinement halted at *R* 0.20 for 3731 data with I>2σ(I). Inspection of data showed a large number with F_o_>>F_c_ indicating coincidental contributions from the other contributing lattice(*s*). A total of 943 reflections which met the two criteria (1) I(obs)/I(calc) > 1.50 and (2) (I(obs)-I(calc)) > 2σ(I(obs)) were then excluded from the dataset. The conventional *R* for these rejected data was 0.47. The ratio criteria (1) was varied down to values of 1.05: although the agreement factors converged at around a ratio of 1.2 (*R* 0.078, for 2252 I>2σ(I) data) no signifcant changes occurred in final su values or parameters compared with the larger dataset. On the basis that another analysis of the data would be possible if the larger dataset was presented, the refinement was continued with the (ratio 1.5) 4280 independent remaining data within the limit of 29° theta. All methyl and tertiary H atoms were refined with *U*_iso_ 1.5 & 1.2 times respectively that of the *U*_eq_ of their parent atom.

### Crystal data

**Table:**

C_18_H_18_N_4_O·CH_2_Cl_2_	*F*_000_ = 816
*M**_r_* = 391.29	*D*_x_ = 1.345 Mg m^−^^3^
Monoclinic, *P*2_1_/*c*	Mo *K*α radiation λ = 0.71073 Å
Hall symbol: -P 2ybc	Cell parameters from 5595 reflections
*a* = 6.8755 (8) Å	θ = 2.4–29.2º
*b* = 16.8913 (17) Å	µ = 0.35 mm^−^^1^
*c* = 16.6677 (18) Å	*T* = 120 (2) K
β = 93.482 (8)º	Block, red
*V* = 1932.1 (4) Å^3^	0.30 × 0.15 × 0.13 mm
*Z* = 4	

### Data collection

**Table:**

Bruker–Nonius APEXII CCD area-detector diffractometer	4280 independent reflections
Radiation source: fine-focus sealed tube	2517 reflections with *I* > 2σ(*I*)
Monochromator: graphite	*R*_int_ = 0.099
Detector resolution: 8.192 pixels mm^-1^	θ_max_ = 29.0º
*T* = 120(2) K	θ_min_ = 2.7º
φ and ω scans	*h* = −9→9
Absorption correction: multi-scan(Blessing, 1995)	*k* = 0→23
*T*_min_ = 0.570, *T*_max_ = 0.955	*l* = 0→22
4280 measured reflections	

### Refinement

**Table:**

Refinement on *F*^2^	Secondary atom site location: difference Fourier map
Least-squares matrix: full	Hydrogen site location: inferred from neighbouring sites
*R*[*F*^2^ > 2σ(*F*^2^)] = 0.095	H-atom parameters constrained
*wR*(*F*^2^) = 0.270	*w* = 1/[σ^2^(*F*_o_^2^) + (0.1824*P*)^2^] where *P* = (*F*_o_^2^ + 2*F*_c_^2^)/3
*S* = 0.99	(Δ/σ)_max_ < 0.001
4280 reflections	Δρ_max_ = 1.36 e Å^−^^3^
237 parameters	Δρ_min_ = −0.47 e Å^−^^3^
Primary atom site location: structure-invariant direct methods	Extinction correction: none

### Special details

**Table:**

Experimental. There were 36108 reflections measured in the data collection (43242 of which 7061 were rejected to 2theta 58 degrees & 73 were systematic absence violations)
Geometry. All e.s.d.'s (except the e.s.d. in the dihedral angle between two l.s. planes) are estimated using the full covariance matrix. The cell e.s.d.'s are taken into account individually in the estimation of e.s.d.'s in distances, angles and torsion angles; correlations between e.s.d.'s in cell parameters are only used when they are defined by crystal symmetry. An approximate (isotropic) treatment of cell e.s.d.'s is used for estimating e.s.d.'s involving l.s. planes.
Refinement. Four low theta angle reflections affected by the backstop were omitted.Refinement of *F*^2^ against ALL reflections. The weighted *R*-factor *wR* and goodness of fit *S* are based on *F*^2^, conventional *R*-factors *R* are based on *F*, with *F* set to zero for negative *F*^2^. The threshold expression of *F*^2^ > σ(*F*^2^) is used only for calculating *R*-factors(gt) *etc*. and is not relevant to the choice of reflections for refinement. *R*-factors based on *F*^2^ are statistically about twice as large as those based on *F*, and *R*- factors based on ALL data will be even larger.

### Fractional atomic coordinates and isotropic or equivalent isotropic displacement parameters (Å^2^)

**Table:**

	*x*	*y*	*z*	*U*_iso_*/*U*_eq_	
Cl1	0.2744 (2)	0.45770 (7)	0.31788 (7)	0.0405 (4)	
Cl2	0.2711 (3)	0.62862 (7)	0.30173 (9)	0.0578 (5)	
O1	0.7615 (4)	0.90197 (14)	0.42000 (15)	0.0176 (6)	
N1	0.7450 (8)	1.0058 (2)	0.6850 (2)	0.0461 (13)	
N2	0.7371 (6)	1.1047 (2)	0.4400 (2)	0.0308 (9)	
N3	0.8028 (7)	0.8020 (2)	0.6872 (2)	0.0382 (10)	
N4	0.7736 (5)	0.41968 (17)	0.40006 (18)	0.0175 (7)	
C1	0.7496 (7)	0.9959 (2)	0.6163 (3)	0.0250 (9)	
C2	0.7542 (6)	0.9836 (2)	0.5329 (2)	0.0187 (8)	
C3	0.7462 (6)	1.0505 (2)	0.4810 (2)	0.0218 (8)	
C4	0.7730 (5)	0.7750 (2)	0.4757 (2)	0.0149 (7)	
C5	0.7705 (6)	0.8175 (2)	0.3964 (2)	0.0160 (7)	
C6	0.7637 (5)	0.9082 (2)	0.5002 (2)	0.0148 (7)	
C7	0.7736 (6)	0.8325 (2)	0.5364 (2)	0.0156 (7)	
C8	0.9567 (6)	0.8083 (2)	0.3533 (2)	0.0234 (8)	
H8A	0.9604	0.8482	0.3107	0.035*	
H8B	0.9611	0.7553	0.3295	0.035*	
H8C	1.0691	0.8154	0.3916	0.035*	
C9	0.5876 (7)	0.8032 (2)	0.3437 (2)	0.0248 (9)	
H9A	0.4733	0.8102	0.3754	0.037*	
H9B	0.5891	0.7492	0.3224	0.037*	
H9C	0.5815	0.8410	0.2990	0.037*	
C10	0.7885 (6)	0.8171 (2)	0.6203 (2)	0.0226 (8)	
C11	0.7752 (6)	0.6928 (2)	0.4885 (2)	0.0186 (8)	
H11	0.7724	0.6756	0.5427	0.022*	
C12	0.7811 (6)	0.6338 (2)	0.4301 (2)	0.0194 (8)	
H12	0.7896	0.6492	0.3756	0.023*	
C13	0.7752 (6)	0.5539 (2)	0.4481 (2)	0.0189 (8)	
H13	0.7644	0.5377	0.5022	0.023*	
C14	0.7848 (6)	0.4965 (2)	0.3880 (2)	0.0174 (7)	
H14	0.8006	0.5143	0.3347	0.021*	
C15	0.7243 (6)	0.3835 (2)	0.4769 (2)	0.0187 (8)	
H15A	0.5982	0.4037	0.4940	0.022*	
H15B	0.8268	0.3944	0.5198	0.022*	
C16	0.7121 (6)	0.2948 (2)	0.4578 (2)	0.0179 (8)	
H16A	0.8381	0.2682	0.4716	0.021*	
H16B	0.6091	0.2690	0.4874	0.021*	
C17	0.6631 (7)	0.2927 (2)	0.3685 (3)	0.0273 (10)	
H17A	0.5222	0.3017	0.3562	0.033*	
H17B	0.7003	0.2413	0.3454	0.033*	
C18	0.7825 (7)	0.3595 (2)	0.3367 (2)	0.0243 (9)	
H18A	0.9184	0.3426	0.3298	0.029*	
H18B	0.7245	0.3793	0.2847	0.029*	
C19	0.2652 (9)	0.5381 (3)	0.2501 (3)	0.0406 (12)	
H19A	0.3774	0.5352	0.2157	0.049*	
H19B	0.1442	0.5349	0.2149	0.049*	

### Atomic displacement parameters (Å^2^)

**Table:**

	*U*^11^	*U*^22^	*U*^33^	*U*^12^	*U*^13^	*U*^23^
Cl1	0.0591 (9)	0.0256 (6)	0.0371 (7)	0.0013 (5)	0.0050 (5)	0.0016 (5)
Cl2	0.1031 (14)	0.0237 (6)	0.0465 (8)	−0.0009 (7)	0.0047 (8)	0.0022 (5)
O1	0.0335 (16)	0.0064 (11)	0.0131 (12)	0.0004 (10)	0.0044 (10)	0.0004 (9)
N1	0.091 (4)	0.024 (2)	0.023 (2)	−0.006 (2)	0.009 (2)	−0.0102 (16)
N2	0.047 (2)	0.0151 (16)	0.030 (2)	−0.0001 (16)	0.0008 (16)	0.0014 (15)
N3	0.063 (3)	0.028 (2)	0.023 (2)	−0.005 (2)	−0.0010 (18)	0.0067 (16)
N4	0.0282 (18)	0.0089 (14)	0.0157 (15)	−0.0017 (12)	0.0042 (12)	0.0002 (11)
C1	0.041 (3)	0.0099 (17)	0.024 (2)	−0.0014 (16)	0.0028 (18)	−0.0020 (14)
C2	0.028 (2)	0.0070 (15)	0.0210 (19)	−0.0003 (14)	0.0015 (15)	−0.0024 (13)
C3	0.030 (2)	0.0104 (17)	0.025 (2)	−0.0013 (14)	0.0018 (16)	−0.0033 (14)
C4	0.0151 (18)	0.0083 (15)	0.0213 (18)	−0.0007 (12)	0.0004 (13)	−0.0016 (13)
C5	0.024 (2)	0.0063 (15)	0.0175 (17)	−0.0030 (13)	0.0010 (14)	−0.0038 (12)
C6	0.0200 (18)	0.0115 (16)	0.0129 (16)	0.0021 (13)	−0.0007 (12)	−0.0012 (12)
C7	0.0216 (19)	0.0112 (15)	0.0141 (16)	−0.0020 (13)	0.0009 (13)	0.0010 (13)
C8	0.033 (2)	0.0152 (17)	0.0230 (19)	−0.0004 (16)	0.0060 (16)	0.0006 (15)
C9	0.042 (3)	0.0128 (17)	0.0189 (19)	−0.0034 (16)	−0.0066 (16)	−0.0003 (14)
C10	0.031 (2)	0.0120 (17)	0.024 (2)	−0.0043 (15)	−0.0023 (16)	−0.0018 (14)
C11	0.023 (2)	0.0105 (16)	0.0226 (18)	−0.0010 (14)	0.0026 (14)	0.0036 (14)
C12	0.024 (2)	0.0100 (16)	0.0238 (19)	−0.0005 (14)	0.0004 (15)	−0.0002 (14)
C13	0.028 (2)	0.0072 (15)	0.0219 (19)	−0.0015 (13)	0.0034 (15)	−0.0007 (13)
C14	0.0207 (19)	0.0081 (16)	0.0233 (19)	0.0021 (13)	0.0020 (14)	0.0019 (13)
C15	0.026 (2)	0.0129 (17)	0.0173 (18)	−0.0016 (14)	0.0023 (14)	0.0008 (13)
C16	0.0220 (19)	0.0093 (16)	0.0221 (18)	0.0018 (13)	−0.0011 (14)	0.0022 (13)
C17	0.040 (3)	0.0142 (18)	0.027 (2)	−0.0056 (17)	−0.0036 (18)	−0.0025 (15)
C18	0.045 (3)	0.0084 (16)	0.0198 (19)	0.0029 (16)	0.0033 (16)	−0.0041 (14)
C19	0.064 (4)	0.036 (3)	0.022 (2)	−0.001 (2)	0.003 (2)	0.0043 (18)

### Geometric parameters (Å, °)

**Table:**

Cl1—C19	1.766 (5)	C9—H9A	0.9800
Cl2—C19	1.753 (5)	C9—H9B	0.9800
O1—C6	1.341 (4)	C9—H9C	0.9800
O1—C5	1.482 (4)	C11—C12	1.397 (5)
N1—C1	1.160 (6)	C11—H11	0.9500
N2—C3	1.141 (5)	C12—C13	1.384 (5)
N3—C10	1.142 (5)	C12—H12	0.9500
N4—C14	1.317 (4)	C13—C14	1.397 (5)
N4—C18	1.471 (5)	C13—H13	0.9500
N4—C15	1.477 (5)	C14—H14	0.9500
C1—C2	1.408 (5)	C15—C16	1.533 (5)
C2—C6	1.388 (5)	C15—H15A	0.9900
C2—C3	1.423 (5)	C15—H15B	0.9900
C4—C7	1.402 (5)	C16—C17	1.505 (5)
C4—C11	1.405 (5)	C16—H16A	0.9900
C4—C5	1.503 (5)	C16—H16B	0.9900
C5—C9	1.510 (5)	C17—C18	1.510 (6)
C5—C8	1.514 (6)	C17—H17A	0.9900
C6—C7	1.412 (5)	C17—H17B	0.9900
C7—C10	1.421 (5)	C18—H18A	0.9900
C8—H8A	0.9800	C18—H18B	0.9900
C8—H8B	0.9800	C19—H19A	0.9900
C8—H8C	0.9800	C19—H19B	0.9900
			
C6—O1—C5	110.0 (3)	C13—C12—C11	122.9 (4)
C14—N4—C18	124.5 (3)	C13—C12—H12	118.6
C14—N4—C15	124.0 (3)	C11—C12—H12	118.6
C18—N4—C15	111.0 (3)	C12—C13—C14	121.2 (4)
N1—C1—C2	179.6 (5)	C12—C13—H13	119.4
C6—C2—C1	121.8 (3)	C14—C13—H13	119.4
C6—C2—C3	119.5 (3)	N4—C14—C13	124.7 (4)
C1—C2—C3	118.7 (3)	N4—C14—H14	117.7
N2—C3—C2	178.8 (5)	C13—C14—H14	117.7
C7—C4—C11	125.2 (3)	N4—C15—C16	103.7 (3)
C7—C4—C5	107.6 (3)	N4—C15—H15A	111.0
C11—C4—C5	127.2 (3)	C16—C15—H15A	111.0
O1—C5—C4	103.0 (3)	N4—C15—H15B	111.0
O1—C5—C9	105.2 (3)	C16—C15—H15B	111.0
C4—C5—C9	113.6 (3)	H15A—C15—H15B	109.0
O1—C5—C8	106.0 (3)	C17—C16—C15	103.6 (3)
C4—C5—C8	113.8 (3)	C17—C16—H16A	111.0
C9—C5—C8	113.9 (3)	C15—C16—H16A	111.0
O1—C6—C2	117.7 (3)	C17—C16—H16B	111.0
O1—C6—C7	110.6 (3)	C15—C16—H16B	111.0
C2—C6—C7	131.7 (3)	H16A—C16—H16B	109.0
C4—C7—C6	108.7 (3)	C16—C17—C18	103.7 (3)
C4—C7—C10	125.4 (3)	C16—C17—H17A	111.0
C6—C7—C10	125.8 (3)	C18—C17—H17A	111.0
C5—C8—H8A	109.5	C16—C17—H17B	111.0
C5—C8—H8B	109.5	C18—C17—H17B	111.0
H8A—C8—H8B	109.5	H17A—C17—H17B	109.0
C5—C8—H8C	109.5	N4—C18—C17	102.5 (3)
H8A—C8—H8C	109.5	N4—C18—H18A	111.3
H8B—C8—H8C	109.5	C17—C18—H18A	111.3
C5—C9—H9A	109.5	N4—C18—H18B	111.3
C5—C9—H9B	109.5	C17—C18—H18B	111.3
H9A—C9—H9B	109.5	H18A—C18—H18B	109.2
C5—C9—H9C	109.5	Cl2—C19—Cl1	111.0 (3)
H9A—C9—H9C	109.5	Cl2—C19—H19A	109.4
H9B—C9—H9C	109.5	Cl1—C19—H19A	109.4
N3—C10—C7	177.6 (4)	Cl2—C19—H19B	109.4
C12—C11—C4	126.9 (4)	Cl1—C19—H19B	109.4
C12—C11—H11	116.6	H19A—C19—H19B	108.0
C4—C11—H11	116.6		
			
C6—O1—C5—C4	1.4 (4)	O1—C6—C7—C4	−1.5 (4)
C6—O1—C5—C9	120.7 (3)	C2—C6—C7—C4	177.8 (4)
C6—O1—C5—C8	−118.4 (3)	O1—C6—C7—C10	176.5 (4)
C7—C4—C5—O1	−2.3 (4)	C2—C6—C7—C10	−4.2 (7)
C11—C4—C5—O1	178.0 (4)	C7—C4—C11—C12	−177.7 (4)
C7—C4—C5—C9	−115.5 (3)	C5—C4—C11—C12	2.0 (6)
C11—C4—C5—C9	64.8 (5)	C4—C11—C12—C13	−177.4 (4)
C7—C4—C5—C8	112.0 (3)	C11—C12—C13—C14	−178.9 (4)
C11—C4—C5—C8	−67.8 (5)	C18—N4—C14—C13	179.6 (4)
C5—O1—C6—C2	−179.4 (3)	C15—N4—C14—C13	8.1 (6)
C5—O1—C6—C7	0.0 (4)	C12—C13—C14—N4	−177.6 (4)
C1—C2—C6—O1	178.0 (4)	C14—N4—C15—C16	175.1 (4)
C3—C2—C6—O1	−1.1 (6)	C18—N4—C15—C16	2.5 (4)
C1—C2—C6—C7	−1.2 (7)	N4—C15—C16—C17	−25.4 (4)
C3—C2—C6—C7	179.7 (4)	C15—C16—C17—C18	38.8 (4)
C11—C4—C7—C6	−177.9 (4)	C14—N4—C18—C17	−151.3 (4)
C5—C4—C7—C6	2.3 (4)	C15—N4—C18—C17	21.1 (4)
C11—C4—C7—C10	4.1 (6)	C16—C17—C18—N4	−36.7 (4)
C5—C4—C7—C10	−175.7 (4)		

### Hydrogen-bond geometry (Å, °)

**Table:**

*D*—H···*A*	*D*—H	H···*A*	*D*···*A*	*D*—H···*A*
C14—H14···N1^i^	0.95	2.52	3.378 (5)	150
C18—H18B···N1^i^	0.99	2.56	3.400 (5)	142

Symmetry codes: (i) *x*, −*y*+3/2, *z*−1/2.
